# Association Between Fresh Embryo Transfers and Frozen–Thawed Embryo Transfers Regarding Live Birth Rates Among Women Undergoing Long Gonadotropin-Releasing Hormone Antagonist Protocols

**DOI:** 10.3389/fcell.2022.884677

**Published:** 2022-04-28

**Authors:** Li Fan, Ni Tang, Chunling Yao, Xiaohua Wei, Yongmei Tang, Jingjing Li, Wenjie Huang

**Affiliations:** Department of Reproductive Medicine, Liuzhou Maternity and Child Healthcare Hospital Affiliated with Women and Children’s Hospital of Guangxi University of Science and Technology, Liuzhou, China

**Keywords:** frozen-thawed transfer, fresh transfer, assisted reproduction, blastocyst, cleavage-stage embryo

## Abstract

**Background:** The availability and use of frozen–thawed embryos after controlled ovarian hyperstimulation for assisted reproduction have increased with improvements in vitrification techniques and the rise of gonadotropin-releasing hormone (GnRH) antagonist protocols. Although evidence has shown that frozen–thawed embryo transfers (FETs) result in higher live birth rates than fresh embryo transfers, it is uncertain whether this association exists in cycles employing the GnRH antagonist protocol.

**Objective:** To test the hypothesis that FETs are more likely to result in a live birth than fresh embryo transfers in a GnRH antagonist protocol cycle and to investigate whether frozen blastocyst transfer increases live birth rates compared to fresh blastocyst transfer.

**Design:** A retrospective historical cohort study was conducted using data collected from the Department of Reproductive Medicine of Liuzhou Maternity and Child Healthcare Hospital for 1,437 patients who underwent the GnRH antagonist protocol between 1 January 2015, and 31 December 2020. The primary outcome was the live birth rate, which was compared between fresh embryo transfer and FET, and the secondary outcomes were clinical pregnancy rate and miscarriage rate, which were compared between the two groups. Analyses were adjusted to account for the age of the patient, number of embryo transfers, day of embryo transfer, and type of infertility.

**Results:** Fresh embryo transfers accounted for 1,026 (71.4%) of the 1,437 patients who underwent the GnRH antagonist protocol in our analysis, while FETs accounted for 411 (28.6%). Patients with fresh and frozen–thawed embryos had comparable median body mass index (body mass index; 22.3 [IQR, 24.6–20.0] vs. 22.0 [IQR, 24.5–19.9]). There was a significant difference in the median age of the fresh embryo transfer group (34.0 [IQR, 39.0–30.0]) and the Frozen–thawed embryo transfer group (32.0 [IQR, 37.0–29.0]). Blastocysts were transferred in 14.6% of the fresh embryo transfer cycles and 45.5% of the FET cycles, whereas they account for 10.4% and 13.0% of all patients, respectively. The mean number of embryos transferred was 2 (IQR, 2.0–1.0) for the fresh embryo transfer group and 1 (IQR, 2.0–1.0) for the FET group, with a significant difference in the mean number of embryos transferred. The live birth rate after fresh embryo transfer vs. FET was 28.7% vs. 34.5% (absolute difference, 5.9%; adjusted relative risk [aRR], 1.15 [95% CI, 0.88–1.51]). The clinical pregnancy rates were 39.9% vs. 46.0%, respectively (absolute difference, 6.1%; aRR, 1.10 [95% CI, 0.85–1.43]). The miscarriage rates were 22.5% vs. 23.8%, respectively (absolute difference, 1.3%; aRR, 1.13 [95% CI, 0.75–1.70]).

**Conclusion:** In this retrospective study of women who underwent assisted reproduction using GnRH antagonists, FETs resulted in a higher live birth rates and clinical pregnancy rates than fresh embryo transfers, which parts of these differences were attributable to embryo stage. However, the interpretation of the findings is limited by the possibility of selection and confounding biases.

## Introduction

Human *in vitro* fertilization (IVF) has become the most common treatment for infertility since its introduction in 1978 (Talaulikar and Arulkumaran, 2013). Since then, numerous assisted reproductive technologies (ARTs) have been developed and refined, including intracytoplasmic sperm injection (ICSI), *in vitro* embryos, ovulation induction, and cryopreservation. Frozen–thawed embryo transfer (FET) was first used in 1983 to avoid embryo replacement in adverse maternal conditions (Trounson and Mohr, 1983). The use of FET has increased rapidly in recent decades (Shapiro et al., 2014), even though the rate of female infertility has remained unchanged over this time (Mascarenhas et al., 2012). Data from European countries in 2013 showed that FET was used in 27.0% of cycles, a 3.9% increase compared to that in 2012, with substantial variation in utilization between countries (Calhaz-Jorge et al., 2017). The same trend has also been reported in the United States (Litzky et al., 2018), China (Shi et al., 2018), and Japan (Ishihara et al., 2014; Takeshima et al., 2016). Couples with non-male factor infertility have had the most substantial increase in the use of FET (from 48.0% to 72.4%) (Takeshima et al., 2016). The justification for using FET in couples with non-female factor infertility is that it can prevent unexpected total unsuccessful embryo transfers and increase the number of embryos or blastocysts, thereby increasing the chances of having a baby. Additionally, it has been reported that FET may also improve perinatal and neonatal outcomes (Sazonova et al., 2012; Wennerholm et al., 2013; Bhattacharya, 2016).

Previous studies have shown that increasing the number of oocytes can improve the cumulative live birth rate (Polyzos et al., 2018; Law et al., 2019). Therefore, increasing oocyte yield with more embryos is crucial for increasing pregnancy rates. The first IVF therapies were conducted in natural unstimulated IVF cycles, but now gonadotrophins are used to induce multiple follicular developments, and gonadotropin-releasing hormone (GnRH) analogs are used to suppress premature luteinizing hormone (LH) surges in IVF. GnRH antagonists competitively block GnRH receptors in the pituitary gland, resulting in the rapid, reversible suppression of gonadotropin secretion and the avoidance of LH (Hall et al., 1988; Huirne et al., 2007), making them a more logical alternative for preventing premature LH surges in IVF. Additionally, the GnRH antagonist protocol has been shown to significantly improve the flexibility and security of clinical applications, and it has been widely utilized in assisted reproductive treatment cycles around the world because of its advantages (Jing et al., 2020).

Although it has been discovered that frozen blastocyst transfers contribute to a higher live birth rate than fresh blastocyst transfers in ovulatory women with good prognoses (Wei et al., 2019), the difference in live birth rates between the FET and fresh transfer groups in GnRH antagonist cycles is still largely unclear. Some cohort studies discovered that FETs resulted in a significantly higher pregnancy rate than fresh embryo transfers (Roque et al., 2015; Chang et al., 2017), whereas other studies discovered that FETs and fresh embryo transfers resulted in similar pregnancy rates, even when a high-quality embryo was transferred during FET (Veleva et al., 2013). Considering that GnRH antagonists are frequently used in clinics with no adverse side effects (Tata et al., 2018) and that different embryonic stages have different reproductive outcomes in fresh embryo transfer cycles (Carvalho et al., 2017), we hypothesize that in GnRH antagonist cycles, FETs are more likely to result in a live birth than fresh embryo transfers, particularly when blastocyst transfer is performed.

## Materials and Methods

### Participants

This retrospective cohort study was conducted at the Department of Reproductive Medicine of Liuzhou Maternity and Child Healthcare Hospital, which is affiliated with the Women and Children’s Hospital of Guangxi University of Science and Technology, and it included all women who underwent controlled ovarian hyperstimulation (COH) and their first IVF/ICSI cycle between January 2015 and December 2020. The following were the inclusion criteria: first IVF/ICSI cycles with at least one embryo available for transfer at either the cleavage or blastocyst stage and planned COH using the GnRH antagonist protocol. The exclusion criteria were cycles with preimplantation genetic testing for aneuploidy (PGT-A), donor oocytes, donor embryos, miscarriages, adverse pregnancy history, uterine anomalies, or incomplete records. Patient information, such as age, infertility diagnosis, infertility type, body mass index (BMI), embryo transfer day, and the number of embryos transferred, was recorded for each cycle.

### COH Protocols

GnRH antagonist protocol: Patients received 150–300 IU/day on day 2 or day 3 of their menstrual cycle until a trigger occurred. The ovarian response was monitored by transvaginal ultrasonography and serum hormone levels, and the r-FSH dosage was adjusted. The patients also received 0.25 mg of a GnRH antagonist (Cetrorelix, Merck Serono, France) daily until the leading follicles reached a mean diameter of 14 mm, which was considered a trigger. When three follicles reached a mean diameter of 17 mm or two follicles reached a mean diameter of 18 mm, 5,000–10,000 IU of recombinant human chorionic gonadotropin (r-HCG, Merck, Darmstadt, Germany) was administered to induce the final maturation of the oocytes. Vaginal ultrasound-guided oocyte retrieval was performed 36 h after HCG rejection. After oocyte retrieval, routine IVF or ICSI was performed based on sperm quality. Luteal phase support was initiated on day 1 after oocyte retrieval by injecting 60 mg of progesterone (Xianju, Zhejiang, China).

### Embryo Transfer

#### Fresh Embryo Transplantation

Embryo transplantation was carried out 3 days after oocyte retrieval or with blastocyst transplantation 5 days after oocyte retrieval. Fresh cycles were canceled if patients had an endometrial thickness <7 mm, severe ovarian hyperstimulation syndrome (OHSS; E2 > 5,000 pg/ml on the trigger day and the number of oocytes acquired >20), no available embryos, or other personal reasons.

#### Frozen Embryo Transplantation

The endometrial preparations in the subsequent FET cycles were programmed using artificial cycles, downregulation + artificial cycles, natural cycles, and induced ovulation cycles, depending on the specific circumstances of the different patients. Artificial cycles were applied to patients with irregular menstrual cycles or polycystic ovary syndrome (PCOS) using exogenous estrogen and progesterone. Oral estradiol (Progynova, Bayer Healthcare, Germany) was administered at 2–3 mg TID (the drug dosage was adjusted according to endometrium thickness), and a transvaginal ultrasound examination was performed 5 days later. GnRH-agonist was used for downregulation in patients with endometriosis, adenomyosis, or uterine fibroids before artificial cycles were applied. An intramuscular injection of Diphereline was used for downregulation when the endometrium was less than 5 mm on day 2 of the menstrual cycle. Thereafter, an artificial cycle was performed after the deregulation standard was attained. Natural cycles were suitable for patients with spontaneous ovulation and regular menstrual cycles. We performed a vaginal ultrasound examination on these patients on days 8–10 of menstruation. An intramuscular injection of 10,000 IU of HCG was administered when the dominant follicles were ≥18 mm and the endometrial thickness was ≥7 mm; FETs were then performed after 3 or 5 days. For ovulation cycles, oral clomiphene or letrozole was administered on day 3 or 5 of their menstrual cycle for a total of 5 days, and HCG was injected to induce ovulation when the dominant follicles were ≥15 mm and the endometrial thickness was ≥7 mm.

The follicle and endometrial thickness were assessed using vaginal ultrasound, and embryo or blastocyst transplantation was performed 3–5 days later under abdominal ultrasound guidance.

### Outcome Measures

In this study, we compared the primary outcome (live birth rate) and the secondary outcomes (clinical pregnancy rate and miscarriage rate) of fresh embryo/blastocyst transfers and frozen–thawed embryo/blastocyst transfers. The primary outcome was defined as the live birth of one or more infants. Clinical pregnancy was defined as an intrauterine gestational sac visible on ultrasound, miscarriage, or termination in the absence of ultrasound data. Miscarriage was defined as pregnancy loss before 20 weeks of gestation.

### Statistical Analyses

All statistical analyses were performed using the Statistical Package for the Social Sciences (SPSS) version 26 (SPSS Inc., Chicago, IL, United States). Medians and interquartile ranges (IQRs) were calculated for baseline and patient characteristics that were not normally distributed. Log-binomial regression models were used to calculate the relative risks (RRs) and 95% confidence intervals (CIs) for the primary and secondary outcomes. Findings from the analyses of secondary endpoints should be interpreted as exploratory because multiple comparisons can produce type 1 errors.

Crude and adjusted analyses were performed to account for the age of the patients, number of embryo transfers, day of embryo transfer, and type of infertility. Sensitivity analyses for the embryo stage for all outcomes. The fresh and frozen–thawed cycles and the embryo stage were evaluated using log-binomial regression interactions. Statistical significance was set at *p* < 0.05.

## Results

### Study Population

**FIGURE 1:**
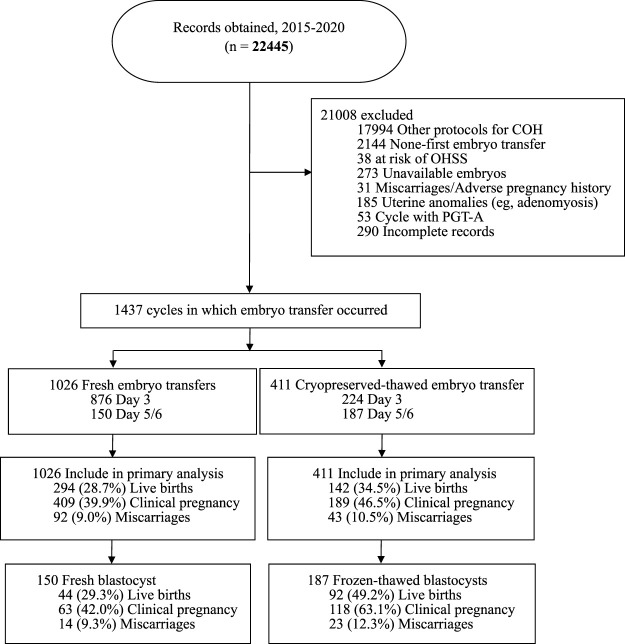
Outline of the selection process, transfer cycle type, embryo stage at transfer and outcomes of embryo transfer cycles included in this study.

Figure 1 shows that 21,008 cycles were excluded for the following reasons: nonavailability of embryo/other COH protocols/not the initial cycle (*n* = 20,411), adverse pregnancy history/miscarriages/uterine anomalies (*n* = 216), PGT-A (*n* = 53), at risk of OHSS (*n* = 38), and incomplete records (*n* = 290). The remaining 1,437 cycles, including 1,026 fresh embryo transfers and 411 FETs, met the eligibility criteria.

### Study Group Characteristics

Patients with fresh and frozen–thawed embryos had comparable median BMIs (22.3 [IQR, 24.6–20.0] vs. 22.0 [IQR, 24.5–19.9]). Patient BMI data were missing in 28 fresh embryo transfer cycles and 10 FET cycles. There was a significant difference in the median age of the fresh embryo transfer group (34.0 [IQR, 39.0–30.0]) and the FET group (32.0 [IQR, 37.0–29.0]). Blastocysts were transferred in 14.6% of the fresh embryo transfer cycles and 45.5% of the FET cycles, whereas they account for 10.4% and 13.0% of all patients, respectively. These data were not normally distributed; the mean number of embryos transferred was 2 (IQR, 2.0–1.0) for the fresh embryo transfer group and 1 (IQR, 2.0–1.0) for the FET group, as presented in Table 1.

**TABLE 1:** Demographics and cycle characteristics of patients.

Characteristics	Fresh embryo transfer (*n* = 1,026)	Frozen-thawed embryo transfer (*n* = 411)	*p*-value
Age, y			
Overall, median (IQR)	34.0 (39.0–30.0)	32.0 (37.0–29.0)	0.001
Age group, No. (%)			
<30	239 (23.3%)	112 (27.3%)	
30–33	239 (23.3%)	115 (28.0%)	
34–37	220 (21.5%)	85 (20.7%)	
38–41	227 (22.1%)	67 (16.3%)	
≥42	100 (9.8%)	32 (7.7%)	
Infertile type^a^			0.004
Primary infertile	351 (34.2%)	174 (42.3)	
Secondary infertile	675 (65.8%)	237 (57.7%)	
BMI			0.14
Overall, median (IQR)	22.3 (24.6–20.0)	22.0 (24.5–19.9)	
Category, No. (%)			
<20	172 (17.3%)	105 (26.2%)	
20–24.9	553 (54.0%)	219 (50.0%)	
25–29.9	237 (23.1%)	66 (14.8%)	
30.0–39.9	35 (3.4%)	10 (2.0%)	
≥40	0	1	
Infertility diagnosis, no. (%)^b^			0.003
Male factor	173 (16.9%)	60 (14.6%)	
Ovulatory	130 (12.7%)	39 (9.5%)	
Tubal factor	689 (67.2)	309 (75.2%)	
Unexplained/other	34 (3.3%)	3 (0.7%)	
Day of transfer, no. (%)^c^			<0.001
Day 3	876 (85.4%)	224 (54.5%)	
Day 5/6	150 (14.6%)	187 (45.5%)	
No. embryos transferred, median (IQR)	2 (2.0–1.0)	1.0 (2.0–1.0)	<0.001
Intracytoplasmic sperm injection, No. (%)^d^	199 (19.4%)	64 (15.6%)	0.09

a Infertility type: primary infertility is infertility occurring in a women who no prior pregnancy; secondary infertility is infertility occurring in a women who have had a previous successful pregnancy.

b Categories for infertile diagnosis: male factor refer to decreased sperm motility, reduced concentration or other issues related to sperm dysfunction that make it difficult to fertilize an oocyte with a sperm under normal conditions; ovulatory infertility refer to conditions that the ovaries are unable to produce oocytes normally; tubal factor refer to the fallopian being damaged or blocked; “unexplained/other” includes patients who have completed an evaluation without obvious explanation for their infertility or patients not meet diagnostic criteria for any other category of infertility.

c Embryo transfer can occur at the cleavage-stage (day 3) or at the blastocyst stage of embryo development (day 5/6). Rarely, a patient may have a day 3 embryo transfer followed by a transfer of a day 5/6 embryo.

d The use of intracytoplasmic sperm injection, in which the sperm is directly injected into the oocyte for fertilization, was performed for either male factor or unexplained infertility as clinically indicated.

Abbreviations: IQR, interquartile range; BMI, body mass index (calculated as weight in kilograms divided by height in meters squared).

Primary infertility was more common in the FET group (42.3% [174/411]) than in the fresh embryo transfer group (34.2% [351/1,026]). There was no significant difference in intracytoplasmic sperm injection utilization between the fresh embryo transfer group (19.4%) and the FET group (15.6%). Meanwhile, we excluded a small number of cycles for which this variable was not recorded (Table 1).

### Primary Outcome

As shown in Table 2, there was a significant difference in the live birth rate after fresh embryo transfer compared to FET (28.7% vs. 34.5%, respectively; absolute difference, 5.9%; unadjusted RR, 1.21 [95% CI, 1.02–1.42]). In addition, after adjusting for patient age, type of infertile, the diagnoses of infertility, and number of embryo transfer did not change the significance of the difference (aRR, 1.33 [95% CI, 1.024–1.716]; data not presented), whereas we observed no significant difference in both live birth rates and clinical pregnancy rates after adjusting for the effect of embryo stage (adjusted RR [aRR], 1.15 [95% CI, 0.88–1.51]), suggesting that parts of these differences were attributable to embryo stage.

**TABLE 2:** Live birth, clinical pregnancy, and miscarriage rates in fresh vs. frozen-thawed embryo transfer cycle.

Outcomes	No. (%)		Absolute difference, %	Relative risk (95% CI)	
	Fresh embryo transfer (*n* = 1,026)	Frozen-thawed embryo transfer (*n* = 411)		Unadjusted	Adjusted^a^
Primary outcome					
Live birth	294 (28.7%)	142 (34.5%)	5.9	1.21 (1.02–1.42)	1.15 (0.88–1.51)
Secondary outcomes					
Clinical pregnancy	409 (39.9%)	189 (46.0%)	6.1	1.15 (1.01–1.31)	1.10 (0.85–1.43)
Miscarriage	92 (22.5%)	43 (23.8%)	1.3	1.01 (0.74–1.39)	1.13 (0.75–1.70)

a Adjusted for patient age, type of infertile, the diagnoses of infertility, number of embryo transfer and embryo stage.

### Secondary Outcomes

After fresh embryo transfer, the clinical pregnancy rate was 39.9%, which was statistically lower than the 46.0% rate after FET (absolute difference, 6.1%; RR, 1.15 [95% CI, 1.01–1.39]). Similarly, there was not statistically significant when the clinical pregnancy rates were adjusted for embryo stage (aRR, 1.10 [95% CI, 0.85–1.43]).

The miscarriage rate was 22.5% after fresh embryo transfer and 23.8% after FET, with no statistically significant difference between the two groups (absolute difference, 1.3%; RR, 1.01 [95% CI, 0.74–1.39]; aRR, 1.13 [95% CI, 0.75–1.70]).

### Cycle Using Blastocyst

The baseline demographics and clinical characteristics of the fresh and frozen cleavage-stage embryo or blastocyst groups are shown in Supplementary eTable S1 and Table 3, respectively. 23.5% of cycles (337/1,437) underwent blastocyst transfer. Of those, 10.4% (150/1,437) were fresh blastocyst transfer group and 13.0% (187/1,437) were frozen-thawed transfer group.

**TABLE 3:** Baseline patient characteristics of women with fresh blastocyst transfers and frozen–thawed blastocyst transfers.

Characteristics (IQR)	No. (%)		*p*-value
	Fresh embryo transfer (*n* = 150)	Frozen-thawed embryo transfer (*n* = 187)	
Age, y	30 (33.0–28.0)	30 (33.0–27.0)	0.743
Infertile type			0.005
Primary infertile	54 (36.0%)	96 (51.34%)	
Secondary infertile	96 (64.0%)	91 (48.66%)	
Infertility diagnosis, no. (%)			0.233
Male factor	30 (20.0%)	33 (17.65%)	
Ovulatory	8.0 (5.33%)	14 (7.49%)	
Tubal factor	109 (72.67%)	140 (74.87%)	
Unexplained/other	3.0 (2.0%)	0 (0.0%)	
No. embryos transferred, median (IQR)	1 (1.0–1.0)	1 (1.0–1.0)	0.016

Abbreviations: IQR, interquartile range.

**FIGURE 2:**
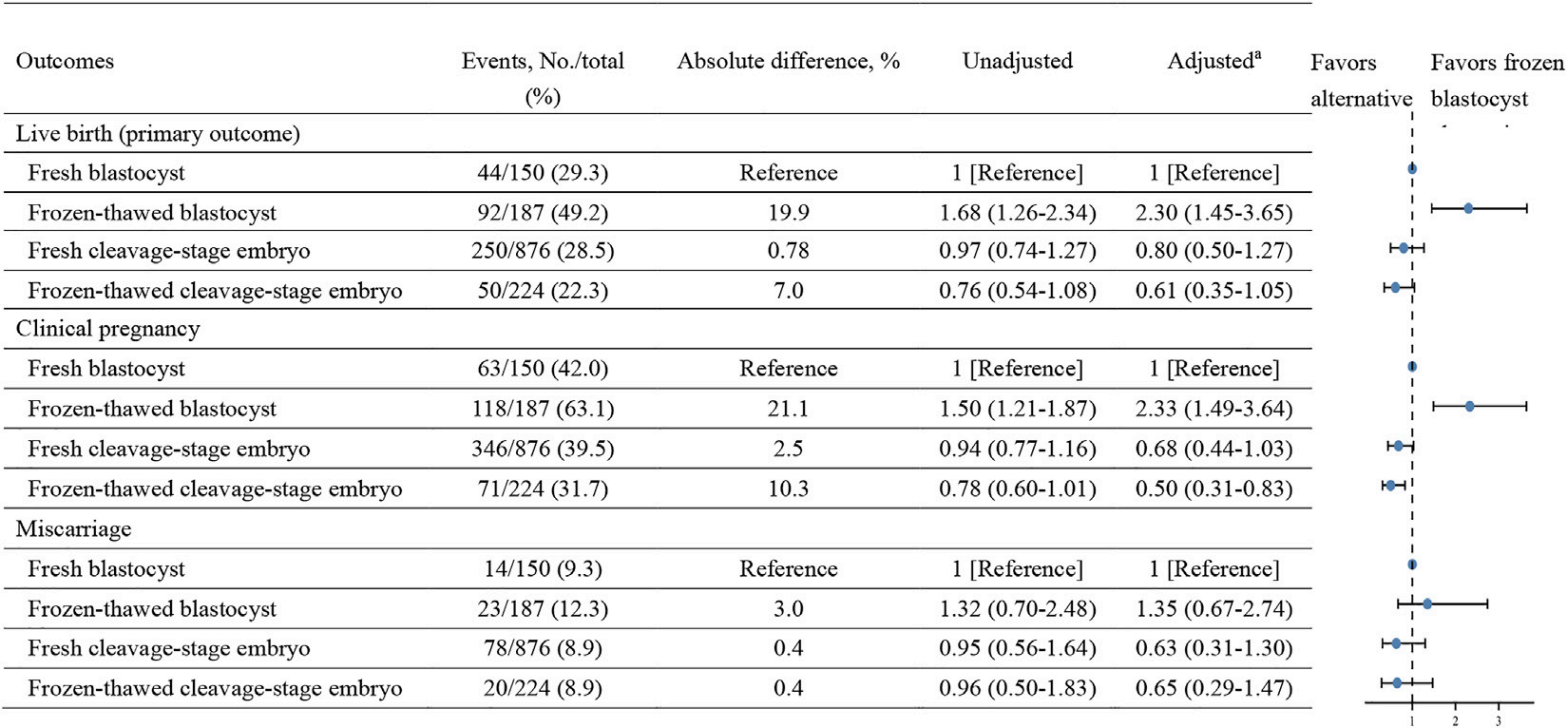
Liver birth, clinical pregnancy, and miscarriage rates in fresh and frozen-thawed embryo transfers with cleavage-stage embryo and blastocyst.

As shown in Figure 2 and Supplementary eTable S3, the FET group had a statistically higher live birth rate for blastocyst transfers than the fresh embryo transfer group (49.2% vs. 29.3%, respectively; absolute difference, 19.9%; aRR, 2.30 [95% CI, 1.45–3.65], which were adjusted to account for the age of patients, type of infertility, diagnosis of infertility, and number of embryo transfers), although the absolute difference in outcomes was smaller than that observed when all fresh embryo transfers were compared to all FETs.

The frozen–thawed blastocyst transfer group also had a statistically significantly higher clinical pregnancy rate than the fresh blastocyst transfer group (63.1% vs. 42.0%, respectively; absolute difference, 21.1%; aRR, 2.33 [95% CI, 1.49–3.65]). There was no significant difference in the miscarriage rate between fresh blastocyst cycles and frozen–thawed blastocyst cycles (9.3% vs. 12.3%, respectively; absolute difference, 3.0%; aRR, 1.35 [95% CI, 0.67–2.74]).

In contrast, live birth rates, clinical pregnancy rates and miscarriage rates did not differ when fresh cleavage-stage embryo transfer group compared with frozen-thawed cleavage-stage embryo group (Supplementary eTable S2). Our results also revealed that there were no statistically significant differences in live birth rates, clinical pregnancy rates, or miscarriage rates between the fresh or frozen–thawed cleavage-stage embryo transfer group and the fresh blastocyst transfer group (Figure 2).

## Discussion

This study demonstrated that, in cycles using the GnRH antagonist protocol, FETs resulted in statistically significantly higher live birth rates than fresh embryo transfer, which parts of these differences were attributable to embryo stage. When comparing fresh blastocyst transfers with frozen-thawed blastocyst transfers, the frozen blastocyst transfer group demonstrated a statistically significantly higher live birth rate and clinical pregnancy rate. This finding is consistent with previous findings that demonstrated higher live birth rates after FET in cycles involving ovarian stimulation.

The proposed mechanisms for improving pregnancy rates using frozen–thawed embryos rather than fresh embryos in GnRH antagonist cycles have demonstrated the possibility that poor endometrial receptivity (ER) is more likely to occur in fresh embryo transfer cycles after controlled ovarian stimulation than in FETs, in which the embryos can be cryopreserved and transferred to a more receptive endometrium (Weinerman and Mainigi, 2014). In addition, the dysregulation of the steroidogenesis-associated gene has been observed in women with endometriosis (Kao et al., 2003; Borghese et al., 2008), suggesting that a disordered endometrium could be exacerbated by high steroid levels, which justifies the FET strategy. Furthermore, using a gestational carrier in fresh embryo transfer or FET can improve IVF outcomes, suggesting that the uterus has a significant effect (Murugappan et al., 2018). Conversely, Insogna et al. (2021) and Miravet-Valenciano et al. (2017) argued that IVF outcomes remain significantly different between fresh embryo transfer and FET cycles despite the recipient uterus not being exposed to superphysiological levels of estrogen or gonadotropins in donor oocyte cycles. Therefore, a disordered endometrium cannot explain the differences observed in the study population.

Embryo quality may be another possible explanation for the advantage of FET over fresh embryo transfer because embryos must be subjected to rigorous selection before freezing (Ginström Ernstad et al., 2019). In contrast, Oktay et al. (2015) suggested that frozen embryo cycles most likely constitute a selection of low-quality embryos because high-quality embryos would have been transferred during the initial fresh transfer. Thus, a prospective cohort study that attempted to control embryo quality by including only initial high-quality embryos that were transferred in fresh or frozen–thawed cycles demonstrated that IVF outcomes can be improved by employing the freeze-all strategy in cases without progesterone elevation (Sacks et al., 2018). Similarly, only the initial transfer in the fresh or frozen–thawed group was analyzed in this study to address the question of embryo quality, and FETs were found to have a higher likelihood of live birth than fresh embryo transfers in patients who used the GnRH antagonist protocol.

Although using blastocysts in fresh cycles is beneficial, it is still uncertain whether or not the stage of embryo development affects live birth and pregnancy rates because they both have advantages and disadvantages (Glujovsky et al., 2016). The *in vivo* environment is likely to be superior to the *in vitro* environment, where a low developmental rate of embryos cultured past the cleavage stage (i.e., only 30%–50% of embryos developed into blastocysts) resulted in a high incidence of cycle cancellation (Marek et al., 1999; Racowsky et al., 2000). In addition, *in vitro* culture that goes beyond embryonic genomic activation can cause embryo damage (Martins et al., 2017). However, the uterine environment is assumed to stress the cleavage-stage embryo because the uterus provides a different nutritional environment from the oviduct (Baart et al., 2006), and late embryo transfer (late on day 3 or early on day 4) is believed to be more analogous to natural cycles (Brown et al., 2016). The morphological criteria used for selecting the highest implantation potential are anticipated to be more accurate than those for selecting cleavage-stage embryos if the culture duration is extended for an additional 2–3 days (days 5–6) (Machtinger and Racowsky, 2013). Indeed, many published studies have shown that fresh blastocyst stage transfers result in higher live birth rates than fresh cleavage-stage transfers (Papanikolaou et al., 2008; Glujovsky et al., 2016; Carvalho et al., 2017). However, our results suggest that fresh blastocyst transfer is not superior to fresh cleavage-stage embryo transfer in terms of live birth or pregnancy rates in women undergoing the GnRH antagonist protocol. This finding corroborated recent findings from a meta-analysis of randomized controlled trials that found no difference in live birth rates, clinical pregnancy rates, or miscarriage rates when blastocyst transfers were compared to cleavage-stage embryo transfers (Martins et al., 2017). Nevertheless, similar to the findings reported by Daimin et al. in a study on ovulatory women (Wei et al., 2019), FET was found to be associated with higher live birth or pregnancy rates in women who underwent the GnRH antagonist protocol when the fresh blastocyst cycles were compared to the frozen–thawed blastocyst cycles, indicating that some physiological differences do exist between fresh and frozen–thawed blastocyst transfers. Although the underlying mechanism is still unclear, the substantial increase in pregnancy rates could be due to a physical change in stiffness in human embryos that were previously cryopreserved (Murayama et al., 2006; Ko et al., 2008; Yanez et al., 2016).

Pregnancy loss cannot explain the differences in live birth and clinical pregnancy rates following fresh embryo transfers or FETs because the miscarriage rates were similar in both groups. Thus, these findings suggest that the frozen–thawed process boosted the implantation potential of embryos obtained from women who had been on the GnRH antagonist protocol. Although there was no difference in miscarriage rates between the fresh and frozen–thawed blastocyst transfer groups, the CIs were large, indicating that further investigation is required.

The findings of this study have several strengths. First, the cost of a successful cycle in the GnRH antagonist protocol is high, ranging from ¥6,757.39 to ¥153,327.20 (Pan et al., 2019). Although the GnRH antagonist protocol is better than other protocols in terms of psychosocial and physical well-being during the first ART treatment (Toftager et al., 2018), these data, considering the significant financial investment, may influence physician recommendation or patients’ decision-making regarding a COH protocol and a fresh embryo transfer vs. FET a priori for convenience. Thus, future research into the cost-effectiveness of fresh embryo transfer vs. FET in this population would be beneficial in guiding practice. Second, recent studies have suggested that the freeze-all policy results in low perinatal morbidity/mortality rates, few birth defects, low birth weight, short gestational age, and additional antepartum hemorrhage (Maheshwari et al., 2012; Roy et al., 2014). However, our findings suggest that frozen–thawed blastocyst transfer results in a significantly higher live birth rate than fresh blastocyst transfer, which may provide useful guidance when deciding whether to pursue a fresh blastocyst transfer or a frozen–thawed blastocyst transfer when using the GnRH antagonist protocol.

However, some limitations must also be considered, particularly its retrospective design, which allows associations between exposures and outcomes to be detected but does not address causality. Second, incomplete data for race and BMI, both of which are known to contribute to miscarriage, may have skewed the results. Additionally, the study was conducted only in a single center, and the sample size was relatively small, limiting the generalizability of the findings. Third, although each clinic’s data were validated by the medical director, it is still possible that certain diseases, such as endometriosis, were underdiagnosed, resulting in women being categorized as having unexplained infertility.

## Conclusion

In this retrospective cohort study of women who underwent ART using the GnRH antagonist protocol, FETs were found to result in higher live birth rates and clinical pregnancy rates than fresh embryo transfers, which parts of these differences were attributable to embryo stage. However, the interpretation of the findings is limited by its retrospective nature. Furthermore, a large, multi-institution collaborative trial is required to determine whether the embryo stage during transfer affects the live birth rate of initial fresh embryo transfers or FETs.

## Data Availability

The raw data supporting the conclusion of this article will be made available by the authors, without undue reservation.
