# Supplementary figures and images for: Association Between Fresh Embryo Transfers and Frozen–Thawed Embryo Transfers Regarding Live Birth Rates Among Women Undergoing Long Gonadotropin-Releasing Hormone Antagonist Protocols
Source: Front Cell Dev Biol. 2022 Apr 28;10:884677. doi: 10.3389/fcell.2022.884677 (PMC9096096; doi:10.3389/fcell.2022.884677)

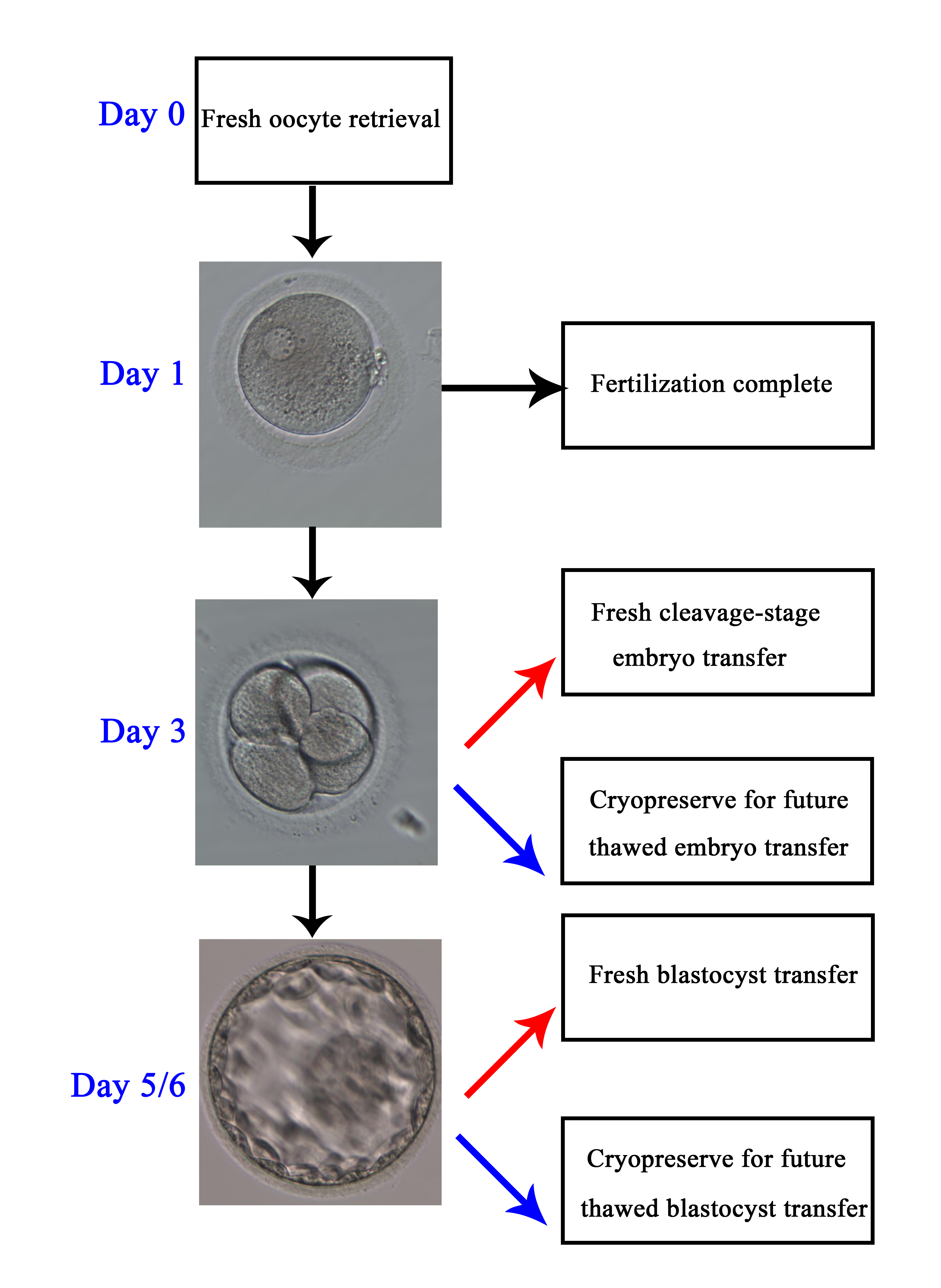

Supplement: Supplementary file 1 [file Image1.TIF]
